# Comparison of balance assessment modalities in emergency department elders: a pilot cross-sectional observational study

**DOI:** 10.1186/1471-227X-9-19

**Published:** 2009-09-28

**Authors:** Jeffrey M Caterino, Rowan Karaman, Vinay Arora, Jacqueline L Martin, Brian C Hiestand

**Affiliations:** 1 The Department of Emergency Medicine, The Ohio State University, Columbus, OH, USA; 2 The Ohio State University College of Medicine, Columbus, OH, USA; 3 The Ohio State University, Columbus, OH, USA

## Abstract

**Background:**

More than one-third of US adults 65 and over fall every year. These falls may cause serious injury including substantial long-term morbidity (due declines in activities of daily living) and death. The emergency department (ED) visit represents an opportunity for identifying high risk elders and potentially instituting falls-related interventions. The unique characteristic of the ED environment and patient population necessitate that risk-assessment modalities be validated in this specific setting. In order to better identify elders at risk of falls, we examined the relationship between patient-provided history of falling and two testing modalities (a balance plate system and the timed up-and-go [TUG] test) in elder emergency department (ED) patients.

**Methods:**

We conducted a cross-sectional observational study of patients ≥ 60 years old being discharged from the ED. Patient history of falls in the past week, month, 6 months, and year was obtained. Balance plate center of pressure excursion (COP) measurements and TUG testing times were recorded. COP was recorded under four conditions: normal stability eyes open (NSEO) and closed (NSEC), and perturbed stability eyes open and closed. Correlation between TUG and COP scores was measured. Univariate logistic regression was used to identify the relationship between patient-provided falls history and the two testing modalities. Proportions, likelihood ratios, and receiver-operating-characteristic (ROC) curves for prediction of previous falls were reported.

**Results:**

Fifty-three subjects were enrolled, 11% had fallen in the previous week and 42% in the previous year. There was no correlation between TUG and any balance plate measurements. In logistic regression, neither testing modality was associated with prior history of falls (*p *> 0.05 for all time periods). Balance plate NSEO and NSEC testing cutoffs could be identified which were 83% sensitive and had a negative likelihood ratio (LR-) of 0.3 for falls in the past week. TUG testing was not useful for falls in the past week, but performed best for more distant falls in the past month, 6 months, or year. TUG cutoffs with sensitivity over 80% and LR(-) of 0.17-0.32 could be identified for these time periods.

**Conclusion:**

Over 40% of community-dwelling elder ED patients report a fall within the past year. Balance plate and TUG testing were feasibly conducted in an ED setting. There is no relationship between scores on balance plate and TUG testing in these patients. In regression analysis, neither modality was significantly associated with patient provided history of falls. These modalities should not be adopted for screening purposes in elders in the ED setting without validation in future studies or as part of multi-factorial risk assessment.

## Background

More than one-third of US adults 65 and over fall every year, sustaining serious injury over 30% of the time [1]. These falls may cause substantial long-term morbidity due to injury-related declines in activities of daily living [2]. Falls are also the leading cause of injury deaths for older adults [3]. This problem will grow as the percentage of the U.S. population 65 years of age and over increases from 12.4% in 2000 to 19.6% in 2030 [4]. Already, approximately 1.8 million emergency department (ED) visits by older adults each year are for falls [3,5]. In addition to those presenting with falls, older ED patients are at an increased risk for falls in the time period around the ED visit [6,7]. As a result, identifying the best method to assess falls risk of elders in the ED has the potential to substantially improve care. In one ED study, one-third of elder falls were due to medical disorders and two-thirds to extrinsic (accidental sources) [8]. Risk factors for falls identified in ED patients have included polypharmacy (79%), home hazards (76%), decreased balance (61%), and arthritis (61%) [9].

Unfortunately, falls risk-assessment is suboptimal in the ED [10,11], and attempted programs have generally been unsuccessful [12,13]. This may be due to a variety of reasons including lack of awareness, complexity of the assessment in a busy ED, and lack of validation of balance assessment modalities in the ED setting and patient population. It is unclear what the best method beyond simple history of falls might be for ED patients. Due to failure of complex falls-risk assessment tools in prior ED studies [13], it is desirable to attempt to identify a single measure. Two modalities for risk assessment that have been described in non-ED settings are the timed-up-and-go (TUG) test and balance plate systems [14-19]. The relationship between these modalities in the ED setting is unclear, as is their relationship to history of falls, which is one of the most significant risk factors for future falling [15]. TUG was chosen because it is the risk-assessment modality recommended by the American Geriatrics Society. The balance plate was chosen due to its portability and ease of use which would allow it to be adopted into the ED setting. Although only one of many possible risk factors in elders for falls, we focused on balance as a measure which could provide readily available data to the ED as distinct from home visits, etc. The primary objective of this pilot study was to compare the associations between falls history, TUG testing, and balance plate assessment in an older ED population. These results will then be available to guide the design of prospective studies to evaluate falls risk-assessments in the older ED population.

## Methods

We conducted a cross-sectional observational study of ED patients at an urban community ED affiliated with an academic medical center. The ED sees approximately 40,000 patients per year and is staffed by board-certified emergency physicians. This study was approved by the hospital's Institutional Review Board and informed consent was obtained. A convenience sample of patients was enrolled between 8 AM and midnight on all days of the week when study investigators were in the department.

Inclusion criteria included: age ≥ 60 years, patient being discharged from the ED, self-reported weight <200 pounds, resident in the community or a personal care home, and self-reported ability to walk 30 feet without help of another person. Use of an assistive device was allowed [7,20]. Patients who presented with a fall remained eligible. Exclusion criteria included: subject incarcerated (in custody of police or prison officials at time of visit), non-English speaking, patient unable to give consent or complete the study tasks, and residence in a nursing home or rehabilitation facility. No memory screening was conducted on the patients.

A patient information sheet and interview were completed upon enrollment. Then, balance plate testing was performed which was followed by administration of a TUG test. Consistent with previous literature, a fall was defined as "any event in which a person inadvertently or unintentionally comes to rest on the ground or another lower level such as a chair, toilet, or bed [21]." Patients with any self reported fall in the previous week, month, 6 months, or year were considered as "fallers" for that time period. The study assessments were performed by two medical students and one undergraduate, all of whom had prior experience in the conduct of clinical research. They did not have specific experience in falls risk-assessment. At least two study personnel were present for each subject. All personnel underwent a 2.5 hour training course sponsored by Bertec personnel on use of the balance plate and demonstrated an ability to use the balance plate to the satisfaction of the Bertec representative. This training also included training in administering the TUG test. For both tests, a step-by-step manual was prepared for reference to ensure the same procedure was followed each time.

The balance plate system used to assess balance and degree of postural sway was the Bertec BalanceCheck Screener™ . The system consists of a 20 × 20-inch platform at ground level connected to a laptop computer. The balance plate detects body sway based on the pressure that the subject's feet apply to the plate surface. Several measures are generated which can be compared to age-adjusted normal values. For testing, each subject stood for 10 seconds under 4 different testing conditions. The first two conditions were eyes open and eyes closed on the balance plate itself, defined as normal stability - eyes open (NSEO) and normal stability - eyes closed (NSEC). These were followed by the patient standing on a 4-inch thick foam rubber pad while on the balance plate. These were labeled as perturbed stability - eyes open (PSEO) and perturbed stability - eyes closed (PSEC). The primary measure assessed by the balance plate for each condition was maximum center of pressure excursion or COP (a distance measured in inches of the major axis of an ellipse calculated along the axis of maximum excursion). The center of pressure is defined as the point on the surface of the plate through which the subject's center of gravity crosses when the subject is motionless. Center of pressure excursion is a measure of postural sway which indicates the magnitude of sway or movement along the long axis of maximum movement.

The TUG test was performed as previously described [20]. Subjects stood up from a chair, walked 10 feet, turned around, walked back to the chair, and sat down. There were no armrests on the chair. If this patient used an assistive device at home, a similar device was provided. The primary measurement was time to complete the entire test.

Means, medians, and proportions were calculated for patient characteristics. An alpha of 0.05 was considered significant. All data was analyzed using Stata, version 10.0 (StataCorp LP, College Station, TX). COP and TUG scores were tested for normality using the Shapiro-Wilk W test. Variables not normally distributed were log-transformed. To assess correlation between COP and TUG scores, the Pearson Correlation Coefficient was calculated for each of the four balance plate testing conditions.

To assess the relationship between the two testing modalities and patient reported history of falls, a series of univariate logistic regression models were constructed with the dependent variable being a fall during the time period in question and the independent variable the COP or TUG score. Time periods examined included the past week, month, 6 months, and year. Significant independent variables were to be inspected for linearity in the logit using LOWESS smoothed scatter plots and appropriate transformations applied as necessary to ensure linearity. Additionally, fractional polynomial analysis was to be used to identify the existence of non-straight-line relationships between the variables. To further define the relationship between the two testing modalities and history of falls, receiver-operator-characteristic (ROC) curves were constructed for each time period and measurement. Area under the ROC curve (AUC) was calculated and sensitivity, specificity, and likelihood ratios reported for likely cutoff values. An area under the curve of 0.5 is considered the point of nondiscrimination, values greater than 0.5 represent increasing discriminatory ability. Sample size was based on an assumed 1 year fall rate of 33%, with 60 patients enrolled this would have provided a total of 20 falls-events. In logistic regression, by the rule of 10 s, 10 events are recommended for each independent variable to be included in the model. As we were planning on only constructing the univariate analyses, 60 patients were expected to provide adequate sample size to detect association [22].

## Results

One hundred and two patients were screened for study entry, 9 (9%) were ineligible and 40 (40%) declined. Fifty-three were enrolled. Reasons for not enrolling included: unable to walk at least 30 feet at baseline (n = 7), unable to understand the consent documents (n = 2), refusal due to acute pain of various body regions (n = 15), and declined with no specific reason given (n = 25). Mean age was 70 years, 70% were female, 70% were black, and 2% were Hispanic. All subjects were able to complete the TUG, NSEO, and NSEC assessments. Three subjects were unable to complete PSEO and PSEC assessments. Falls were reported among 11% in the past week (95% confidence interval [CI] 4-23%), 23% in the past month (95% CI, 12-36%), 34% in the past six months (95% CI, 22-48%), and 42% in the past year (95% CI, 28-56%). Patients were included in all time periods after the most recent fall. For example, all patients falling in the past week were also considered to have fallen in the past month, 6 months, and year. No patient had a presenting complaint of a fall. Mean TUG score was 16.4 seconds (standard deviation 6.1 seconds)

Neither COP nor TUG tests were normally distributed so these results were log transformed. The correlation coefficient between logTUG and the logCOP of each of the balance plate testing conditions was: NSEO 0.15, NSEC 0.05, PSEO 0.10, and PSEC 0.11, indicating poor correlation between the two testing modalities.

In the univariate logistic regression models, there was no significant relationship between the dependent variables of patient reported falls and the independent variables of logCOP or logTUG. The coefficients and odds ratios for these regression models are shown in Table 1. As logCOP and logTUG were not significantly associated with any falls outcome in the models, LOWESS smoothed plots were not constructed and fractional polynomial analysis was not performed. Although not noted in Table 1, PSEO and PSEC testing were also non-significantly related to falls at all time periods.

**Table 1:** Results of the univariate regression models comparing testing modalities with patient reported falls

	**β coefficient**	**Standard error of coefficient**	**Odds ratio**	**95%CI of odds ratio**	***p*-value**
**Falls in past week**					
logCOP NSEO	1.31	1.11	3.71	0.42-32.95	0.239
logCOP NSEC	0.68	1.11	1.96	0.22-17.30	0.541
logTUG	0.29	1.27	1.33	0.11-16.13	0.821
**Falls in past month**					
logCOP NSEO	-0.30	0.91	0.74	0.12-4.43	0.739
logCOP NSEC	0.04	0.96	1.04	0.16-6.82	0.965
logTUG	2.21	1.15	9.14	0.96-87.40	0.055
**Falls in past 6 months**					
logCOP NSEO	-0.56	0.77	0.57	0.13-2.58	0.466
logCOP NSEC	0.83	0.83	2.29	0.45-11.56	0.317
logTUG	1.18	0.94	3.25	0.51-20.50	0.210
**Falls in past year**					
logCOP NSEO	-1.14	0.82	0.32	0.06-1.59	0.164
logCOP NSEC	1.08	0.87	2.94	0.54-16.11	0.214
logTUG	1.15	0.96	3.15	0.48-20.71	0.233

NSEO = normal stability, eyes open; NSEC = normal stability, eyes closed; COP = center of pressure; TUG = timed-up-and-go; CI + confidence interval

To further analyze the relationship between history of falls and the testing modalities, ROC curves for prediction of fall were constructed for the balance plate COP measurements and TUG. AUCs were then calculated (Table 2). For measurements with an AUC of ≥ 0.60, proportions and likelihood ratios at various cutoffs were examined to identify useful cutoff values (Table 3). Balance plate testing was only useful for the NSEO and NSEC components, and these were most sensitive for falls in the past week. TUG testing was not useful for identifying patients with falls in the past week (AUC 0.47) but performed better for more distant falls in the past month, 6 months, or year. As noted in Table 3, there were several cutoffs with negative likelihood ratios of approximately 0.30, indicating a small decrease in the likelihood of falls in the setting of a negative test. For TUG these included values ranging from 12-15 seconds depending on the time period studied.

**Table 2:** Diagnostic performance of testing modalities for predicting falls using area under the receiver-operator-characteristic curve analysis*

	**Time period of the fall**							
**Measurement**	**Past week**		**Past month**		**Past 6 months**		**Past year**	
NSEO	0.65	(0.46-0.84)	0.57	(0.36-0.77)	0.52	(0.35-0.70)	0.47	(0.31-0.64)
NSEC	0.63	(0.37-0.88)	0.54	(0.38-0.71)	0.58	(0.42-0.74)	0.60	(0.44-0.76)
PSEO	0.50	(0.20-0.79)	0.45	(0.25-0.64)	0.42	(0.24-0.59)	0.43	(0.27-0.60)
PSEC	0.42	(0.12-0.72)	0.46	(0.25-0.66)	0.56	(0.38-0.73)	0.56	(0.40-0.73)
TUG	0.47	(0.26-0.67)	0.69	(0.54-0.83)	0.66	(0.51-0.80)	0.64	(0.48-0.79)

*Values are reported as area under the receiver-operator-characteristic curve with 95% confidence intervals

NSEO = normal stability, eyes open; NSEC = normal stability, eyes closed; PSEO = perturbed stability, eyes open; PSEC = perturbed stability, eyes closed; TUG = timed-up-and-go

**Table 3:** Test performance for predicting falls of balance testing modalities at optimal cutoff values

**Measure**	**Cutoff value**	**Sensitivity (%)**	**Specificity (%)**	**Positive LR**	**Negative LR**
NSEO - past week	0.31	83	55	1.86	0.30
NSEC - past week	0.42	83	53	1.78	0.31
NSEC - past year	0.33	86	35	1.34	0.38
TUG - past month	14 sec	92	49	1.79	0.17
TUG - past month	15 sec	83	58	2.00	0.28
TUG - past 6 months	12 sec	94	34	1.44	0.16
TUG - past 6 months	14 sec	83	51	1.72	0.32
TUG - past year	12 sec	91	35	1.41	0.26

NSEO = normal stability, eyes open; NSEC = normal stability, eyes closed; TUG = timed-up-and-go; LR = likelihood ratio; sec = seconds

Given reports of underreporting rates of past falls of up to 20% [23], we sought to determine what effect underreporting might have. For the TUG test, we assumed that the highest 5 values of TUG among patients reporting no falls in the past year actually represented an unreported fall based on past reports of an association between TUG and falling [20]. When conducting the univariate analysis for 1 year falls under this assumption, the AUC for TUG increased from 0.64 to 0.79 with 81% sensitivity and 61% specificity at a cutoff of 12 seconds.

## Discussion

In this study of older adults being discharged from the ED, we found that over 40% reported falling within the past year. This high percentage was reported in a cohort in which no patient presented with a fall-related complaint, and is consistent with rates reported in other studies of community-dwelling elders [21]. It demonstrates the importance of continued efforts to find effective and usable falls risk-stratification tools for older ED patients. Previous studies have largely concentrated on patient questionnaires and comprehensive geriatric assessment instruments [12,13,24,25]. Many have used additional staff with geriatrics expertise, a resource not available in most EDs [24,25]. These attempts have met with varying degrees of success. Those utilizing only ED personnel have generally been unsuccessful, likely due to failure of ED staff to follow the protocol suggestions [12,13]. As a result, future efforts should concentrate on finding modalities acceptable to and adaptable by most EDs. These would ideally be rapidly and easily implemented. For example, the TUG test requires no additional equipment and can be performed by any trained ED personnel. The balance plate requires a modest initial investment, but could be adopted in EDs if purchased by them. The plate is mobile and can be kept on a small cart. It does not require recalibration with moving. The time to complete both tests in our study, although not specifically measured, was approximately 2-3 minutes.

Our goal was perform a pilot study analyzing the relationships between several potential falls risk-assessment modalities in the ED setting. Patient-supplied history of falls is only one of several potential risk factors for future falls and may provide an incomplete picture of risk of future falls [15]. As comprehensive review of all falls risk factors is unlikely to occur in the ED setting, identifying easily administered and interpretable testing modalities is crucial. The first steps in assessing such modalities include assessing their ability to be completed in the ED. In our study, both balance plate and TUG tests were obtainable in the ED as all patients were able to complete the TUG test and all but three were able to complete balance plate testing.

The second step is to understand the relationship between the modalities. If results differ between modalities, further study would be required of all of them. Conversely, if results do not vary, future studies could concentrate on only one. In our ED population, there was minimal correlation between TUG and balance plate results. This may be due to the different components of balance measured by the two modalities as TUG measures dynamic balance and the balance plate measures static balance. Other studies have noted only moderate association between dynamic and static balance in elders [26]. In fact, balance assessment modalities measuring different constructs may be complementary [17]. As a result, further study should clarify the advantages, if any, of complementary testing as compared to selecting a single modality in the ED.

Balance plates using limits of stability measurements have been used to predict fall risk in both institution-dwelling and community-dwelling elders [18,19,27,28]. In addition to the lack of correlation between balance plate and TUG testing, there was no relationship between the balance plate testing and patient provided history of falls in univariate logistic regression analysis. The balance plate NSEO and NSEC measures did have an AUC of >0.60 in identifying falls in the week prior to ED visit. For these measures, cutoffs could be identified with a sensitivity >80% which were somewhat useful in ruling out a fall within the past week with a negative likelihood ratio of approximately 0.3. However, specificity was low and the confidence intervals for the ROC curves were wide, limiting the conclusions that may be drawn from them and indicating that few patients would be judged to be at low risk of falls.

An additional concern limiting conclusions to be drawn from our use of the balance plate was the decision to proceed with a single assessment of each balance plate test. Several authors have noted that multiple repeat sessions may be required to obtain the most reliable intra-session measurements and best correlation between measurements when performing balance plate testing [29,30]. We chose a single measurement for two reasons. First, it is the recommended regimen from the balance plate manufacturer. Second, the test is most useful in the ED if it is short and easily accomplished. Repeat measurements would tend to decrease the usability of the test in the ED. However, given our results, it appears that a single session of COP measurements may not provide useful information in the ED setting.

The TUG test is recommended as a quick, routine falls-screening modality for older patients [15,31,32]. It is easy to perform, has demonstrated high intra-tester and inter-rater reliability [14], has shown construct validity [14,16,33], does not require specialized personnel, and is recommended by current guidelines [15]. In this ED study, TUG test results were related neither to balance plate testing nor to patient self-reported history of falls. In the regression models, the only near-significant relationship was between TUG testing and falls within the past 6 months. AUC for patient report falls was generally poor and with wide confidence intervals. The AUC was greatest for falls within the past month, 6 months, or year. For these time periods, TUG cutoffs could also be identified with a negative likelihood ratio sufficient to provide a small to moderate decrease in posttest likelihood of fall. The optimum TUG cutoffs of 12-15 seconds we found are consistent with those of other studies in community-dwelling elders [20]. Again, however, the results of the regression analysis and the wide CIs of the ROC curves indicate that there is generally poor agreement between TUG and patient reported falls history. In a study conducted among ED patients, Walker et al found that the TUG test was poorly predictive of ED revisit or admission, further supporting its lack of a proven role in ED patients [34].

The lack of association between TUG and falls history in the ED is different than previous reports in community-dwelling elders where TUG was able to discriminate between those with a history of falls and non-fallers, correctly classifying approximately 70% of patients [35]. In another study, TUG had a high sensitivity and specificity of 87% in predicting past falls [20]. It may be that in the acutely-ill ED setting, the TUG test has different test characteristics than in other community-dwelling elder populations. Based on out results and the results of Walker et al [34], the TUG test should not be adopted for ED use without validation in this specific population either alone or as part of a multifactorial risk assessment model.

Our study was limited by the fact that, although eligible, no patients presenting with a fall were included in the study cohort. Most previous studies of balance assessment have occurred in such patients, and this high-risk group is the recommended target for balance assessment [15]. ED patients who present with a fall have been shown to have worse performance on dynamic and static balance testing than non-fallers [7]. It may be that studying these modalities in elders presenting to the ED with a fall will improve the test characteristics.

We did not classify falls and did not focus on patients with known risk factors for falling. As a result, our cohort may have been at particularly low risk of falling and this may have affected our results. The possibility of a Type II error may also have occurred due to the size of the sample studied. Relying on patient recall may have resulted in missed episodes of falling. Similar self-reporting has previously been proven valid, with 80-89% sensitivity and 90-95% specificity for recall of a fall at 1 year in a review of 6 studies of falls recall [23]. However, these studies have not been conducted in acutely-ill ED patients, raising the possibility of even greater rates of misreporting. The possibility of at least 20% underreporting may have influenced the negative association in our study as noted by the example of TUG testing in the results section. In the worst case, assuming that those with the highest TUG scores had failed to report their falls, there was a substantial increase in AUC for the TUG test. Therefore, prospective evaluation of future falls would be the ideal method to identify an association between these tests and falling. We did examine prior falls at various time periods in our models given the acute nature of most ED visits as it is unclear if testing in acutely-ill ED patients will have similar characteristics to that conducted in stable outpatients. Additionally, we did not gather specific data on time taken to complete the tests which would be of interest prior to adoption in the ED. Most importantly, prior to applying these testing modalities in the ED, it will take further prospective trials to determine if these can reliably predict falls after the ED visit, and if acting on that information will be of benefit.

## Conclusion

In conclusion, over 40% of community-dwelling elder ED patients report sustaining a fall within the past year. Balance plate and TUG testing were feasibly conducted in an ED setting. There is no relationship between scores on balance plate testing and the TUG test in these patients. Both modalities also have limited overlap with patient provided history of falls. As each may be providing different information, future studies of falls risk-assessment in older ED patients should test several modalities and screening questions to determine the optimal method to screen for future falls.

## Competing interests

The authors declare that they have no competing interests.

## Authors' contributions

JMC conceived of and designed the study, analyzed the data, and drafted the manuscript. RK, VA, and JLM participated in the design of the study, enrolled patients and administered the study interventions, and helped to draft the manuscript. BCH participated in the design of the study and performed the greater part of the statistical analysis. All authors read and approved the final manuscript.

## Pre-publication history

The pre-publication history for this paper can be accessed here:


